# Effects of systemic Anatolian propolis administration on a rat-irradiated osteoradionecrosis model

**DOI:** 10.1590/1678-7757-2023-0231

**Published:** 2023-10-27

**Authors:** Sefa ÇOLAK, Aras ERDIL, Fikret GEVREK

**Affiliations:** 1 Tokat Gaziosmanpaşa University Faculty of Dentistry Department of Oral and Maxillofacial Surgery Tokat Turkey Tokat Gaziosmanpaşa University, Faculty of Dentistry, Department of Oral and Maxillofacial Surgery, Tokat, Turkey.; 2 Uşak University Faculty of Dentistry Department of Oral and Maxillofacial Surgery Uşak Turkey Uşak University, Faculty of Dentistry, Department of Oral and Maxillofacial Surgery, Uşak, Turkey.; 3 Tokat Gaziosmanpaşa University Faculty of Medicine Department of Histology and Embryology Tokat Turkey Tokat Gaziosmanpaşa University, Faculty of Medicine, Department of Histology and Embryology, Tokat, Turkey.

**Keywords:** Osteoradionecrosis, Propolis, Radiotherapy, Rats

## Abstract

**Objective:**

Radiotherapy after head and neck cancer is associated with the risk of osteonecrosis development. This study aims to investigate the effectiveness of systemic propolis application to prevent the disease as it has no definite treatment protocol despite the proposed treatment methods and significantly decreases individuals’ quality of life.

**Methodology:**

In total, 29 male Wistar-Albino rats were divided into control, 35 Gy irradiation (Group 1), 35 Gy irradiation+100 mg/kg/ml propolis administration (Group 2), and 35 Gy irradiation+200 mg/kg/ml propolis administration groups (Group 3). Propolis was first applied on the day after radiotherapy, except for the control group. Right first and second molars were extracted from all rats three weeks following radiotherapy. Samples were collected seven weeks after radiotherapy. Osteoblast and osteoclast counts were calculated by histomorphometric analysis. Immunohistochemical analysis determined bone morphogenic protein-2 (BMP-2) and transforming growth factor beta-3 (TGFβ-3).

**Results:**

Group comparison found non-significant differences regarding osteoblast (p=0.130) and osteoclast (p=0.063) counts. However, Group 1 showed the lowest mean osteoblast (OBL: 82.63 [±13.10]) and highest mean osteoclast counts (OCL: 12.63 [±5.55]). OBL/OCL ratio showed significant differences between groups (p=0.011). Despite the significant difference between the Control and Groups 1 (p=0.006) and 2 (p=0.029), Group 3 showed a non-significant difference (p=0.091). For BMP-2 and TGFB3, the control group showed significant differences with the other two groups (p<0.001), except for Group 3.

**Conclusion:**

Anatolian propolis showed beneficial effects in a radiotherapy-mediated osteonecrosis model, highlighting its potential as a promising intervention.

## Introduction

Radiotherapy (RT) is used to treat head and neck malignancies, often with chemotherapy and surgery. Although it can effectively manage head and neck malignancies, it can also lead to complications, such as xerostomia, increased susceptibility to infections, alopecia, mucositis, radiation caries, and mandible or maxilla osteoradionecrosis (ORN).^1^ ORN may occur after RT as a late sequelae, especially in cases in which total radiation doses exceed 60 Gy on local traumas such as uncontrolled periodontal disease, incompatible prostheses, and tooth extraction.^2^ ORN lesions consist of persistent necrotic bone tissue exposure for over three months in a previously irradiated area with negative metastatic bone disease or tumor recurrence history.^3^ The incidence of ORN is reported to vary from 5 to 15% after head and neck radiotherapy. Many patients suffer from the symptoms of ORN, including impaired wound healing, pain, malodor, infection, trismus, orocutaneous fistulae, exposed necrotic bone, and even pathological fractures, particularly in the oral and maxillofacial region.^4^

Fractionated doses of ionizing radiation accumulate in tumoral tissues and cause death or senescence of rapidly dividing malignant cells. Despite the high sensitivity of ionizing radiation in target tissues, cell damage in adjacent healthy tissues cannot be prevented entirely, often affecting adjacent bone tissues.^5^ Ionizing radiation affects osteoblast function and proliferation, such as collagen production and induces cell cycle arrest.^6^ Since osteoblasts are essential for proper osteoclast differentiation and functional bone homeostasis, the effects of RT on them affect osteoclast metabolism, at least indirectly.^7^ While studies indicate that osteoclast numbers decrease or remain stable after RT, opposite results have also been reported, causing a lack of clarity.^4,8^

The pathophysiology of ORN has been investigated for many years and different theories have been proposed. Currently, it is suggested that a radiotherapy-induced fibroatrophic process forms ORN. In its primary stage, radiotherapy-induced endothelial cells cause edema, leading to necrosis and local microvasculature ischemia, destruction of endothelial cells, and chronic non-specific inflammation (which is characterized by increased vascular permeability with vascular thrombosis). As the process continues, abnormal fibroblastic activity and extracellular matrix irregularity are effective. Finally, in the fibroatrophic phase, myofibroblasts undergo apoptosis and a small number of cells, dense extracellular matrix, and fibroatrophy develop in the tissues. The effects of this fibroatrophic process on bone tissue due to RT have been observed for many years and are rarely reversible.^4^

Anatomical differences in the oral cavity suggest that the mandible shows a greater risk of ORN than the maxilla. The mandible has limited vascular support (usually within the RT area), whereas the maxilla contains a dense source of vascular anastomosis outside the RT area. Moreover, intense mineralization of the mandible increases doses of absorbed radiation.^9^

Propolis, also known as bee glue, is a natural non-toxic substance honey bees produce by mixing the secretions of their hypopharyngeal glands with the digested resins they collect from plant leaves or trees.^10^ Propolis is a natural mixture with more than 300 identified components to date, such as phenolic acid, cinnamic acid, caffeic acid, aromatic aldehydes, alcohols, amino acids, vitamins, various esters, and flavonoids.^10,11^ It has been used in anti-inflammatory, antibacterial, wound healing, and burn treatments in different geographical regions since ancient times. Today, it has been shown to have biological activities such as antibacterial, antiviral, fungicidal, anti-inflammatory, antioxidant, hepatoprotective, immunomodulatory, tumoricidal, and radioprotective effects.^11^ The literature claims that, as an anti-inflammatory agent, propolis inhibits prostaglandin synthesis, activates the thymus gland, triggers the immune system by inducing phagocytosis, stimulates cellular immunity, and increases healing in epithelial tissues.^12^

To our knowledge, no study has evaluated the effects of propolis on an experimental mandibular ORN model. Since ORN is challenging to treat, prevention has great importance.^13^ Considering the biological effects described above, this study aims to evaluate the effectiveness of Anatolian propolis on mandibular bone tissue in a proven ORN model. The null hypothesis of this study posited that administering Anatolian propolis would fail to significantly prevent or reduce the severity of radiotherapy-induced mandibular osteoradionecrosis (ORN) in an experimental rodent model.

## Methodology

### Experimental animals

In total, 29 young-adult male Wistar Albino rats were included in this study. Their weight ranged from 300 to 350 g and they were obtained from and housed in the Experimental Medical Research Unit of Tokat Gaziosmanpasa University under controlled temperature (21 [±1] ^0^C) and humidity (50% [±10%]) with a 12-h light and dark cycles and offered access to pelleted rodent diet and water ad libitum. The methodology of this study was reviewed and approved by the Local Ethical Committee of Animal Trials of Tokat Gaziosmanpasa University under approval number 51879863 - 190. The protocol of this study was carried out per the Tokat Gaziosmanpaşa University Animal Experiments Local Ethics Committee Establishment and Operational Directive and the Helsinki Declaration Ethical Laws on Animal Experiments.

The required sample size was calculated based on a previous study on the mandibular ORN model following RT to ensure accurate results.^14^ Using a one-way analysis of variance test, a minimum of 24 animals were needed to detect an effect size of 1 with a 95% power. To account for potential animal losses during the trial, the sample size was increased by 20%, totaling 29 animals. The rats were randomly divided into four groups, with five individuals in the control group and eight in every three experimental groups. RT was applied to the head and neck of the rats in all experimental groups. The surgical and radiation techniques used in this study were based on a previously successful model of radiation-induced mandibular ORN in rats.^15^

### Irradiation procedures

**Figure 1:**
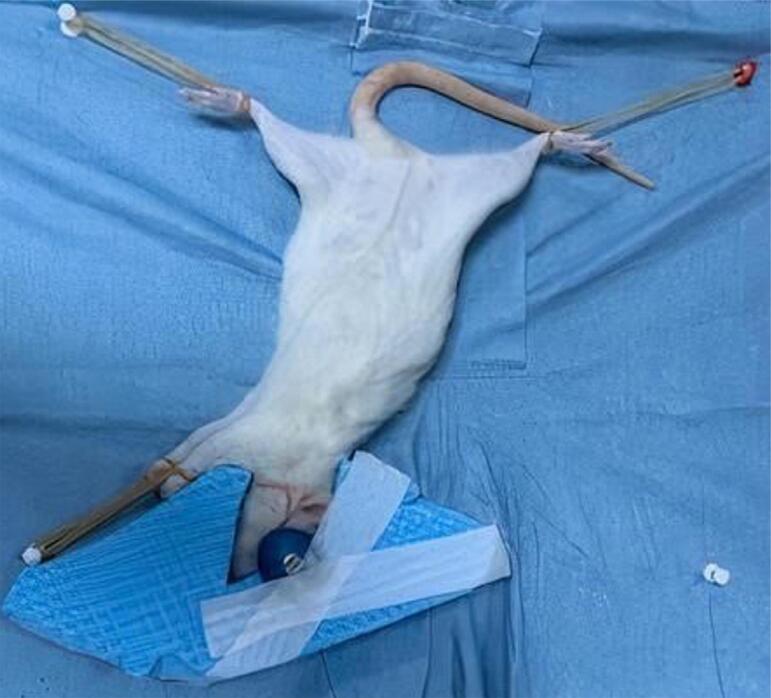
The illustration depicts how a rat was secured during radiotherapy

Radiotherapy was administered under the supervision of a radiation oncologist at Tokat Gaziosmapaşa University, Department of Radiation Oncology, with a Varian Clinac DHX 5776 Linear Accelerator (LINAC) (Varian Medical Systems, Palo Alto, CA, U.S.A). Irradiation was performed under general anesthesia with intraperitoneal (i.p.) injections of ketamine (60 mg/kg) and xylazine (3 mg/kg). The rats were positioned on their left sides and stabilized. To avoid exposing their brains and eyes, the target volume was set with the three-dimensional conformal radiotherapy technique from two areas using 6 MV photon energy (Figure 1). In total, 24 rats in the experimental groups were positioned in the same manner and a single radiation dose of 35 Gy was administered at the rate of 2.5 Gy/min.

### Propolis delivery and surgical procedures

Following the day of irradiation, a water-soluble, high caffeic acid-containing propolis extract (Bee’o Water-Soluble Propolis Droplets, SBS Scientific Bio Solutions Inc., Istanbul, Turkey) delivery began by daily oral gavage at a dose of 100 mg/kg/ml in Group 2 and 200 mg/kg/ml in Group 3. The atraumatic extraction of the first and second right molars was performed under general anesthesia (injections of ketamine (60 mg/kg) and xylazine (3 mg/kg), i.p.) three weeks after irradiation. The rats were euthanized seven weeks after irradiation by cervical dislocation after a high-dose anesthetic injection.

### Tissue sample acquisition and tracking

The mandibles were removed as a whole and fixed directly in buffered neutral 4% formalin solution for three days. Following fixation, the samples were washed all day long under running water, dehydrated in increasing series of alcohols (70, 80, 90, 96, and 100%, respectively), pellucid in xylenes, and after impregnation in three separate paraffin melts at 600 °C, they were blocked by embedding in paraffin blocks in the same orientation and horizontally on their long axis. Consecutive thin serial sections of 5-µm thickness were obtained from the blocked mandible with a rotary microtome (Leica RM٢١٣٥, Leica Biosystems, Deer Park, IL, U.S.A.). These tissue sections were placed on slides and prepared for microscopic analysis by histochemical and immunohistochemical staining.

### Hematoxylin-Eosin staining

The sections embedded in paraffin blocks were kept in an oven at 60 °C, deparaffinized by passing them through xylenes for 3 × 5 minutes and a series of gradually decreasing concentrations of alcohol (100, 96, 90, 80, and 70%, respectively), and hydrated by plunging them into distilled water.

Sections were kept in hematoxylin for 10-15 minutes, washed under running water for 5 minutes, immersed in acid alcohol, and after being washed again under running water, were kept in eosin dye solution for 3 minutes and taken to distilled water, which was changed several times to remove excess dye. The sections were immersed in 80 and 90% alcohol, respectively, in 96% alcohol for 1 minute, and in absolute alcohol for 2 minutes. After being kept in xylenes for 3 × 5 minutes, Entellan (Merck, Darmstadt, Germany) was dripped onto the tissue sections on the slide and covered with a coverslip.

### Osteoblast and osteoclast cell index analyses

Hematoxylin-eosin-stained mandibular bone tissue preparations were examined with a research light microscope (Nikon Eclipse Ti2, Nikon Europe, Amstelveen, The Netherlands) by a blinded histologist unaware of the study groups. For the analyses, osteoblasts and osteoclasts were counted separately in an average of five or six consecutive sections from each mandible and in five randomly different areas in each section with an average of 100 cells in total. Software integrated into the microscope was used for cell counts (NIS-Element, Nikon Europe, Amstelveen, The Netherlands). Osteoblast and osteoclast cell counts and ratios were calculated for each mandible.

### Immunohistochemical analyses

Tissue sections with 5-µm thickness from the formalin-fixed and paraffin-embedded tissues were taken to slides and stained according to bone morphogenic protein-2 (BMP-2) and transforming growth factor beta-3 (TGFβ-3) indirect immunohistochemical staining protocol.

The tissue sections were kept in an oven at 60 °C and rehydrated by lowering alcohol content (100, 90, 80, and 70%, respectively) and immersed into distilled water after deparaffinization in xylene (3 × 5 minutes). After antigen retrieval in a microwave in 10 mM citric acid, the sections were incubated in a 3% hydrogen peroxide (H_2_O_2_) solution for 10 minutes. The sections were washed in phosphate-buffered saline (PBS) for 3 × 5 minutes, limited with a hydrophobic (pap pen) pen, and left for 15 minutes in a humid dark environment by dripping non-immune-blocking serum. After removing the blocking agent, BMP-2 and TGFβ-3 primary antibodies (1:100; Santa Cruz Biotechnology, Heidelberg, Germany) were added to the sections and incubated at 0 [±4] °C overnight in a closed humid environment. After the primary antibody incubation was completed, the sections were washed with PBS for 3 × 5 minutes and incubated with biotinylated secondary antibody (goat IgG) (IHC Select, Merck, Darmstadt, Germany) for 45 min at room temperature in a closed, humid, and dark environment. To incubate another secondary antibody — streptavidin-horseradish peroxidase-conjugated reagent (HRP) (EMD Millipore Corporation, Burlington, MA, U.S.A.) —, the sections were washed with PBS for 3 × 5 minutes and the antibody was dropped and incubated for 30 minutes in a closed humid environment. For coloring, an amino ethyl carbazole (AEC) chromogen substrate solution (Merck) was dripped onto the sections that were washed with PBS for 3 × 5 minutes and observed after 5-10 minutes. Sections that were immersed in distilled water were counterstained with Hematoxylin, a water-based (aqueous mount reagent) closure solution was dripped onto them, and the sections were closed with a coverslip. PBS was leaked onto several sections instead of the primary antibody to create negative control sections.

To determine immunostaining intensities, calculations were made using the immunostaining intensity scoring criteria in Table 1 in the NIS-Element software (Nikon Europe) at 40 X magnification with a light microscope (Nikon Europe). The unstained and stained cells with BMP-2 and TGFβ-3 were counted categorically regarding their staining reaction intensities (Table 1). The weighted average results obtained from the groups were converted to Histoscore (H - score) values with the formula [ ∑Pi(i+1) ] (In the formula; i: the staining intensity score, Pi: the percentage of stained cells).

**Table 1:** Immunohistochemical Staining Intensity Scores

Score	Intensity Class
0	Negative Staining
1+	Weak Positive
2+	Moderate Positive
3+	Strong Positive

### Statistical analyses

Statistical analyses were performed on GraphPad Prism Version 8.2.1 (GraphPad Software, San Diego, CA, U.S.A.). The normal distribution of data was evaluated by the Shapiro-Wilk test. Homogeneity of variances was also checked. The differences between the countable variables (BMP-2, TGFβ-3, Osteoblast and Osteoclast counts, and Osteoblast/Osteoclast ratios) of the study groups were evaluated by one-way analysis of variance (ANOVA) test if distributed normally and by the Kruskal-Wallis test if distributed abnormally. Intergroup differences were evaluated with Tukey’s HSD (following ANOVA tests) and Dunn’s (following Kruskal-Wallis tests) multiple comparisons tests. P values below 0.05 were considered statistically significant.

## Results

This study included 28 rats. However, one rat from the Control group passed away due to a possible anesthetic overdose before its teeth were extracted. Macroscopically, this study found no clinical signs of ORN in any group except Group 1 and collected tissue samples.

### Histomorphometric findings

After hematoxylin-eosin staining, this study analyzed such sections to count their osteoblasts and osteoclasts. The Shapiro-Wilk test showed that osteoblast numbers significantly deviated from normal distribution. Consequently, the data underwent analysis using the Kruskal-Wallis test, which showed non-significant differences between groups (p=0.130). However, osteoblast numbers were the highest in the unirradiated Control group (n=4, 93.25 [±2.75]) and smallest in Group 1 (n=8, 82.63 [±13.10]). Also, Group 2 showed an 89.88 [±2.53] mean osteoclasts and Group 3, 90.63 [±2.61].

**Figure 2:**
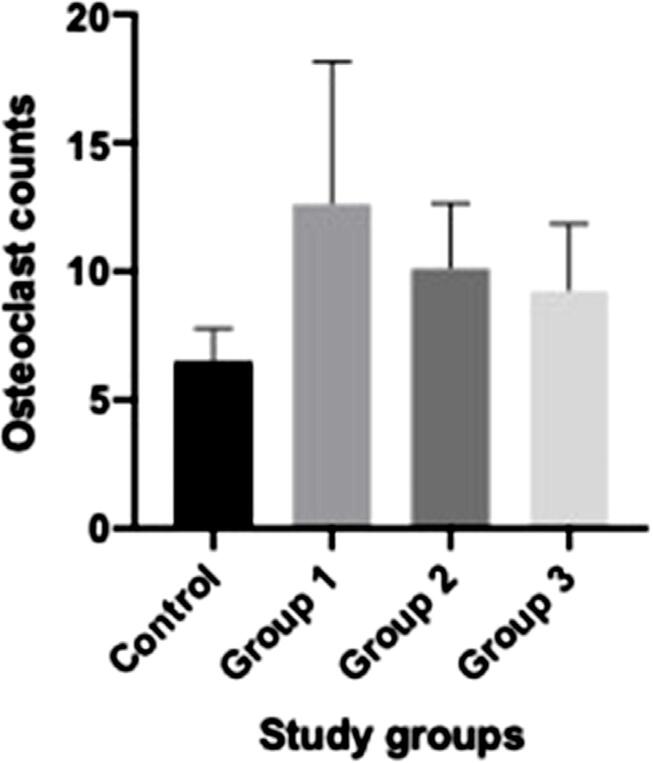
Graphical comparative display of osteoclast numbers regarding the study groups

**Figure 3:**
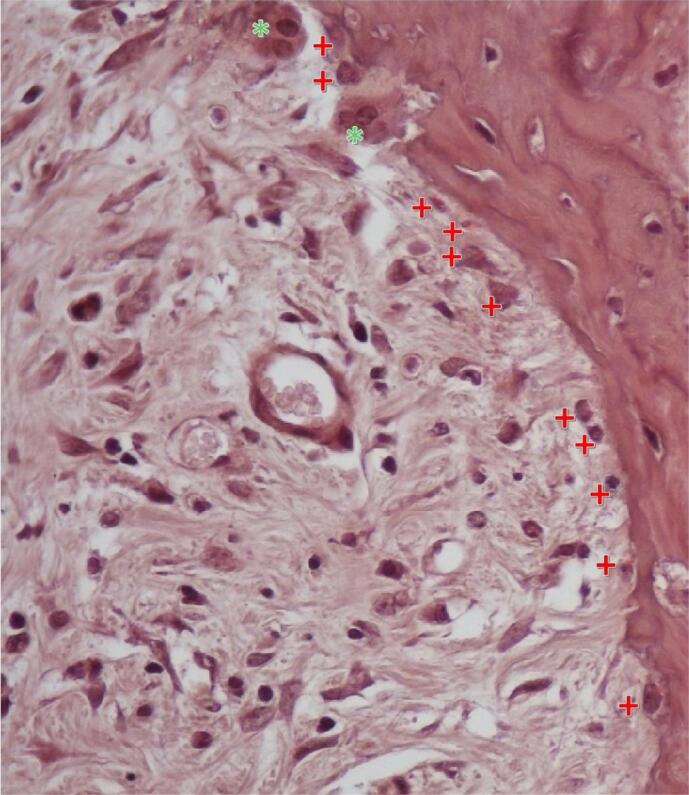
A representative image of cell count by NIS-Element, indicating the osteoblasts and osteoclasts on a sample. * donates osteoclasts, + donates osteoblasts. (Hematoxylin-eosin stained, Scale: 20µm)

Osteoclast numbers, showing normal distribution, had marginal differences according to one-way ANOVA (p=0.063). The lowest osteoclast numbers were in the Control group (6.5 [±1.29]). Irradiated groups (Group 1: 12.63 [±5.55], Group 2: 10.13 [±2.53], and Group 3: 9.25 [±2.60]) showed higher osteoclast counts than the Control group. This study obtained a value close to a significant difference due to the significant difference between the Control group and Group 1 in the Tukey’s HSD test (p=0.049) (Figure 2). Furthermore, the noted variation led to a significant difference in osteoblast(OBL)/osteoclast(OCL) ratios between groups, as observed in the intragroup analysis (one-way ANOVA) results (p=0.011). While this study found a significant difference between the Control group (15.84 [±5.71]) and the means of Group 1 (8.08 [±3.55]) and Group 2 (9.44 [±2.40]), Group 3 showed no significant differences (10.58 [±3.04], p=0.091) (Table 2). Figure 3 shows the representative microscopic histomorphometric view of osteoblast and osteoclast count.

**Table 2:** The intergroup comparisons of osteoblast/osteoclast ratios

Groups (n)	Mean	Standard Deviation	Standard Error	Median (IQR)	95% Confidence Interval Lower bound Upper Bound		p Value
Control Group (n=4)	15.84	5.71	2.86	15.43	6.74	24.94	0.006*
Group 1 (n=8)	8.08	3.55	1.25	8.54	5.11	11.05	0.862#
Group 2 (n=8)	9.44	2.40	0.85	9.77	7.43	11.45	0.029+
Group 3 (n=8)	10.58	3.05	1.07	10.35	8.03	13.12	0.914†

Intragroup comparison was analyzed by ANOVA, intergroup comparisons were analyzed by Tukey’s HSD, and indicated with characters *, #, +, †. Control Group: Non-radiated group, Group 1: 30 Gy radiated, Group 2: 30 Gy radiotherapy+100mg/kg/ml propolis, Group 3: 30 Gy radiotherapy+200 mg/kg/ml propolis. Bold p values indicate significant intergroup differences.

* indicates the comparison between Control Group – Group 1

# indicates the comparison between Group 1 – Group 2

+ indicates the comparison between Control Group – Group 2

† indicates the comparison between Group 2 – Group 3

### Immunohistochemical findings

**Figure 4:**
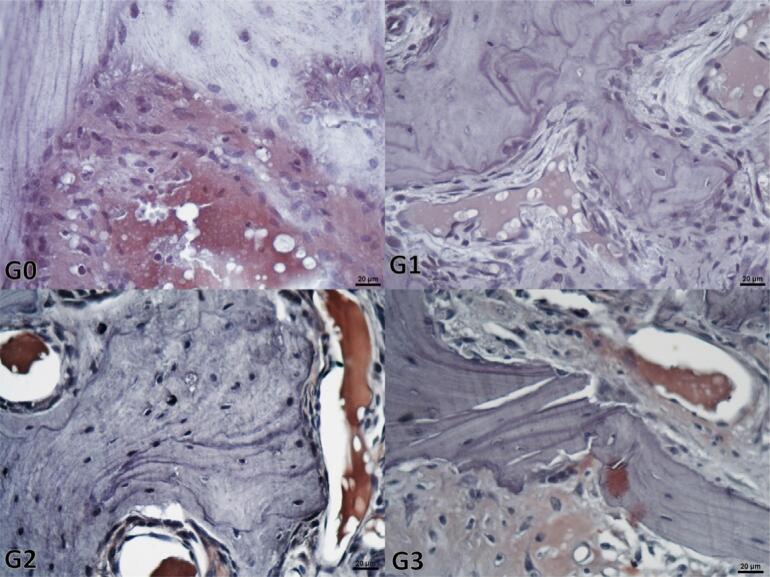
Representative view indicating the immunohistochemical BMP-2 staining of the samples from the study groups. The most intense staining is in the G3 groups and the least intense staining is in G1, whereas the intensity between these two refers to the G0 and G2 groups. (G0: Control group, G1: 30 Gy radiotherapy, G2: 30 Gy radiotherapy+100mg/kg/ml propolis, G3: 30 Gy+200 mg/kg/ml propolis) (Scale bar: 20µm)

**Figure 5:**
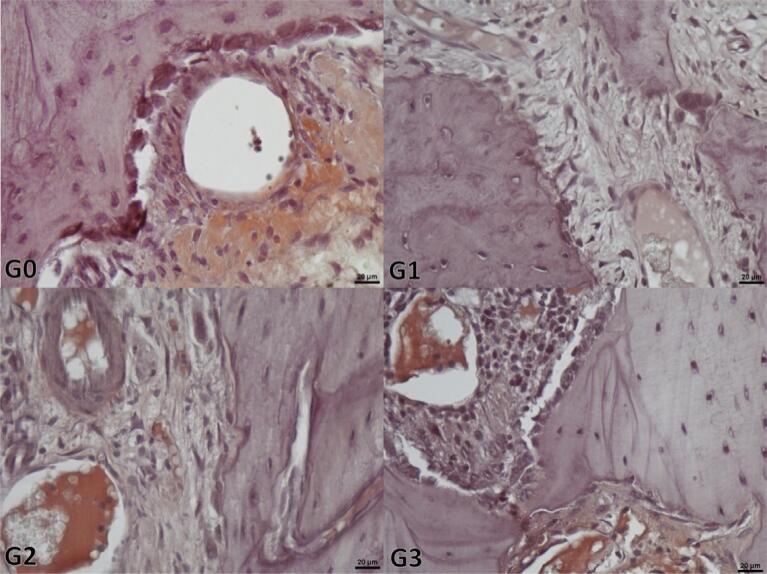
Representative view indicating the immunohistochemical TGFβ-3 staining of the samples from the study groups. The most intense staining is observed in the G3 groups, and the least intense staining is in the G1, while the intensity is between these two in the G0 and G2 groups. (G0: Control group, G1: 30 Gy radiated, G2: 30 Gy radiotherapy+100mg/kg/ml propolis, G3: 30 Gy+200 mg/kg/ml propolis) (Scale bar: 20µm)

The analysis of the H-scores of TGFβ-3 (p<0.001) and BMP-2 (p<0.001) showed significant differences between groups. While this study found significant differences between Group 1 (p<0.001) and the other groups, there were non-significant differences between the Control group, Group 2 (p=0.970 for BMP-2, p=0.477 for TGFβ-3), and Group 3 (p=0.994 for BMP-2, p=0.517 for TGFβ-3) for both variables (Tables 3 and 4). Figures 4 and 5 show the representative microscopic views of the study groups regarding the staining intensities of TGFβ-3 and BMP-2.

**Table 3:** The intergroup comparisons of bone morphogenic protein-2 (BMP-2) H-scores

Groups (n)	Mean	Standard Deviation	Standard Error	Median (IQR)	95% Confidence Interval Lower bound Upper Bound		p Value
Control Group (n=4)	234.4	17.62	8.80	229.6	206.4	262.4	<0.001*
Group 1 (n=8)	134.9	33.22	11.75	140.4	107.1	162.7	<0.001#
Group 2 (n=8)	228.3	16.08	5.68	229	214.9	241.7	<0.001+
Group 3 (n=8)	230.9	14.93	5.28	235.6	218.4	243.4	0.995†

Intragroup comparison was analyzed by ANOVA, intergroup comparisons were analyzed by Tukey’s HSD and indicated with characters *, #, +, †. Control Group: Non-radiated group, Group 1: 30 Gy radiated, Group 2: 30 Gy radiotherapy+100mg/kg/ml propolis, Group 3: 30 Gy radiotherapy+200 mg/kg/ml propolis. Bold p values indicate significant intergroup differences.

* indicates the comparison between Control Group – Group 1

# indicates the comparison between Group 1 – Group 2

+ indicates the comparison between Group 1 – Group 2

† indicates the comparison between Group 2 – Group 3

**Table 4:** The intergroup comparisons of tissue growth factor beta-3 (TGFβ-3) H-scores

Groups (n)	Mean	Standard Deviation	Standard Error	Median (IQR)	95% Confidence Interval Lower bound Upper Bound		p Value
Control Group (n=4)	185.7	38.17	19.09	192.9	124.9	246.4	0.047*
Group 1 (n=8)	136.1	36.62	12.95	145.6	105.5	166.7	<0.001#
Group 2 (n=8)	211.6	12.59	4.45	210.2	201.1	222.1	<0.001+
Group 3 (n=8)	210.4	27.72	9.80	211.7	187.2	233.6	0.999†

Intragroup comparison was analyzed by ANOVA, intergroup comparisons were analyzed by Tukey’s HSD and indicated with characters *, #, +, †. Control Group: Non-radiated group, Group 1: 30 Gy radiated, Group 2: 30 Gy radiotherapy+100mg/kg/ml propolis, Group 3: 30 Gy radiotherapy+200 mg/kg/ml propolis. Bold p values indicate significant intergroup differences.

* indicates the comparison between Control Group – Group 1

# indicates the comparison between Group 1 – Group 2

+ indicates the comparison between Group 1 – Group 3

† indicates the comparison between Group 2 – Group 3

## Discussion

This study aimed to investigate the impact of systemically administered Anatolian propolis on tooth extraction-induced osteoradionecrosis in an animal model with head and neck radiotherapy. This study evaluated the levels of BMP-2 and TGFβ-3 molecules and monitored osteoblast and osteoclast counts to gain insights into the potential protective effects of Anatolian propolis on ORN. Based on our results, the groups treated with Anatolian propolis showed statistically significant increases in bone regeneration markers than the irradiated group that received no propolis. In contrast, this study found no similar results for osteoblast and osteoclast counts. While this research observed non-significant results in intra-group comparisons, it found the mean of the highest osteoblast and lowest osteoclast counts in the unirradiated control group. However, cell counts were higher in the 200 mg/kg propolis-administered group than in the other irradiated study groups. The null hypothesis stating that Anatolian propolis would fail to have a significant protective effect on the mandibular ORN has been rejected based on these outcomes.

Despite the current advances in radiotherapy techniques, the high ORN incidence rates (37%) reported in the 1970s could only be reduced to 5%. Although the literature recommends methods such as PENTOCLO (pentoxifylline, tocopherol, and clodronate), hyperbaric oxygen, and bone-containing free flap reconstructions following mandibulectomy to treat ORN, it still lacks a consensus on a treatment method.^16^ Considering the complexity of treatment and patients’ systemic health, it is essential to prevent the disease before it occurs, if possible.^3^ To prevent ORN, using hyperbaric oxygen therapy (HBO), antibiotic prophylaxis, pentoxifylline-tocopherol, and ultrasound has been suggested. However, the fact that HBO therapy causes complications such as middle ear barotrauma, myopia, and (more rarely) pneumothorax and arterial air embolism and is contraindicated in chronic obstructive pulmonary diseases, ill-controlled chronic heart failure, and active tumors limits its prophylactic use. Antibiotic prophylaxis for prevention is controversial since infection is unnecessary to form the condition according to the currently accepted fibroatrophic theory on pathophysiology. The efficacy of approaches such as ultrasound and pentoxifylline-tocopherol combination, which have shown beneficial effects in managing ORN, are still being investigated. Furthermore, methods such as HBO, ultrasound, and pentoxifylline-tocopherol may require multiple weeks to produce noticeable effects.^16^

Based on our results, systemic propolis administration showed promising effects on the development of mandible ORN. The beneficial effects of propolis have also been shown in previous models of rapid maxillary expansion,^17^ distraction osteogenesis,^18^ ovariectomy-mediated osteoporosis,^19^ dental trauma,^20^ and fractures in rats.^21^ The aforementioned studies drew attention to the anti-inflammatory properties of propolis. Similarly, Guler Avcı, et al.^22^ (2022) reported that propolis has radioprotective effects on radiotherapy-mediated oral mucositis and tongue damage. The positive effects of propolis in these different models are associated with its high flavonoid content.^17^ A study comparing the flavonoid contents of varying propolis species showed that Anatolian propolis from the Euro-Siberian phytogeographic region is particularly rich in pinocembrin, a flavonoid compound.^23^

In addition to its anti-inflammatory effects, that propolis affects the osteoclasts and osteoblasts responsible for bone remodeling.^23^ Pileggi, et al.^25^ (2009) investigated the effects of propolis on murine macrophages and mouse bone marrow cells and showed that it could reduce osteoclast-like cells. Another study showed propolis to reduce tartrate-resistant acid phosphatase-positive cells from human peripheral blood mononuclear cells.^26^ However, this study found the lowest mean osteoclast count in the non-irradiated control group, the highest mean value in the radiated group without propolis, and a significant difference between them. This study found no significant differences with the other propolis-administered study groups. This outcome disagrees with the data in the literature but it should be noted that this study is methodologically different. Although the fibroatrophic theory is generally accepted in the pathogenesis of osteoradionecrosis (valid today), it was thought that the osteoclastic nature of the disease might show this result.^3^ Moreover, Freitas, et al.^27^(2017) investigated the radioprotective effects of black grape juice and found higher osteoclast counts in the whole brain of irradiated rats than non-irradiated samples, despite administering the active substance.

Propolis has been shown to promote osteoblast differentiation by increasing the expressions of runt-related transcription factor 2, osterix, osteocalcin, and type 1 collagen alpha.^28^ Regarding osteoblast numbers, although this study found no statistically significant difference, counts were higher in nonirradiated and propolis-treated radiated groups. It was also observed that the number of osteoblasts was proportional to the increased applied propolis concentration. This result agrees with Tolba, El-Sefari, and Omar’s^29^ (2017) conclusions that caffeic ester phenyl ester (CAPE), a propolis component, can increase osteoblast activation and number at increasing doses in a dexamethasone-mediated osteoporosis model. However, this study found significant differences in the calculated osteoblast/osteoclast ratios and observed no significant difference between the control group and the group that received radiation and propolis at a dose of 200 mg/ml/kg.

BMP-2 and TGFβ-3 are members of the same TGFβ growth factor family and are known to play a role in bone regeneration.^30^ Radiotherapy-mediated reduction of BMP-2 in mandibular bone tissue was shown in osteoradionecrosis models in rodents.^31^ Also, it has been reported that early secretion of TGFβ-3 in mandibular alveolar defects will increase osteoprogenitor cell migration in the defect area.^32^ Due to these characteristics, this study also investigated differences between groups. Results showed no significant differences between the propolis-administered study groups and the Control group by significant differences between all other three groups and the radiated group. Although no study has using the same model and growth factors in the literature, Somsanith, et al.^33^(2018), in their research with propolis-loaded titanium oxide nanotube implant design, observed that BMP-2 levels and osseointegration were significantly higher in propolis groups.

Despite the reported results, this study has several limitations. First, the lack of a previous study may be hinder comparisons. Therefore, a validated method in ORN formation^15^ was preferred to evaluate the effectiveness of systemic propolis administration. It is important to note that research has neither evaluated the impact of propolis on the development of osteoradionecrosis in humans nor determined ideal treatment dosages. This offers a significant limitation. However, the reported median dose of propolis (2-7.3 g/kg) that is lethal in rats corresponds to the range of 1.4-70 mg/kg/day for humans, indicating that the propolis doses in this study are relatively safe.^34^Furthermore, this study primarily derived its findings from immunohistochemical and histomorphometric methods. While these techniques offer valuable insights, the limitations of this study include the absence of alternative approaches, such as enzyme-linked immunosorbent assay (ELISA), to assess various bone regeneration markers and their expression levels. Additionally, this study ignored osteogenesis-related gene expressions using real-time polymerase chain reaction (PCR), which could have provided further comprehensive data.

Moreover, future studies should investigate the ORN preventive effects of propolis at different doses within the safe range with human participants and the effects of propolis on genetic pathways in osteoradionecrosis. This study evaluated the effectiveness of propolis after radiotherapy. Considering that the CAPE molecule, a propolis component, has cytotoxic effects on oral cancer cells,^35^ the effectiveness of applications during radiotherapy should also be investigated.

## Conclusion

Despite its limitations, this study showed the encouraging effects of Anatolian propolis on bone regeneration markers BMP-2 and TGFβ-3 in a radiotherapy-induced osteoradionecrosis model. These initial results warrant further investigation by high-quality clinical studies to comprehensively assess the potential protective effects of propolis to prevent the development of ORN *in vivo*.
